# TLR-Mediated Preterm Birth in Response to Pathogenic Agents

**DOI:** 10.1155/2010/378472

**Published:** 2010-08-23

**Authors:** Jessica E. Thaxton, Tania A. Nevers, Surendra Sharma

**Affiliations:** Department of Pediatrics, Women and Infants' Hospital of Rhode Island—Warren Alpert Medical School of Brown University, Providence, RI 02905, USA

## Abstract

The incidence of preterm birth in developed countries has risen in the past decades. Underlying causes for this enigmatic pregnancy complication are numerous, yet infectious agents that induce dysregualtion of immunity at the maternal-fetal interface pose one of the most probable causes of preterm birth. This paper highlights two factors regarding maternal infections that trigger unscheduled inflammatory sequences that are deleterious to the maternal-fetal balance necessary to maintain pregnancy. Firstly, we discuss the role of Toll-like receptors (TLRs) as sentinels of uterine immunity in the context of response to pathogens. We highlight the idea that particular TLR activations lead to differential immune cascades that induce preterm birth. Secondly, two alternative routes of pathogenic entry may prove to be critical for inducing preterm birth via a cytokine storm or a secondary and currently unknown cell-mediated mechanism of uterine inflammation. This paper summarizes pathways that underlie activation of adverse and diverse immune responses to foreign agents that may result in preterm birth.

## 1. Introduction

Healthy pregnancy is the result of a tightly regulated system of crosstalk between maternal and fetal structures. Implantation and parturition are specifically characterized by states of inflammation [1, 2]. Proinflammatory cytokines, matrix degrading proteins, altered transcriptional factors, rapid hormonal changes, and immune cell activity are paramount for uterine activation and the onset of labor [3]. In contrast, the gestation period, comprised of decidualization, placentation, and fetal development, requires uterine quiescence guided by high levels of progesterone and the production of anti-inflammatory cytokines from both maternal and fetal cells [4, 5]. Due to the immediacy with which the onset of labor takes place and the resultant necessary shift from anti- to proinflammatory signal cascades, it is not surprising that unscheduled parturition is the most high risk state of pregnancy for adverse outcomes.

Preterm birth can result from a range of causes such as exposure to environmental triggers, maternal stress, fetal or maternal genetic abnormalities, or hormonal imbalance. However, infection is one of the most heralded causes of preterm birth due to the drastic link between underlying infectious agents and their ability to promote inflammatory responses [6–8]. It is well documented that the vast majority, upwards of 90% of preterm births that occur before gestational week 28 can be correlated to the presence of infectious agents and severe inflammation [9]. Furthermore, a significant number of placentas obtained from preterm deliveries show pathological signs of chorioamnionitis which can result from a number of differential pathogenic agents [10]. Thus, while the evidence for infection-mediated preterm birth is substantial, the underlying mechanisms that induce early birth in response to pathogenic presence remain vague.

Investigation into the mechanisms that lead to preterm birth in response to pathogenic agents should take into account several key factors. Firstly, the route of entry that a given foreign agent takes determines where the agent will ultimately subsist and what pathways will be activated. Recent evidence demonstrates that the same pathogen, delivered through alternative routes, can lead to differential inflammatory responses ([11–13], our unpublished results). Therefore, the mechanisms that underlie initiation of preterm birth may be dependent on the organ or tissue where a pathogen enters. Secondly, different pathogens may elicit disparate inflammatory responses. Data from humans and animal models show that alternative sets of cytokines coupled with activation of maternal or fetal cells are triggered in response to different pathogenic agents, yet all result in unscheduled inflammation [14, 15]. A strong explanation for initiation of distinct immune pathways is probably the activation of toll-like receptors (TLRs). TLRs are a diverse set of innate immune sentinel receptors highly conserved throughout evolution. Each TLR1–10 is specific for a different pathogen associated molecular pattern (PAMP) [16]. Importantly, TLRs are highly expressed at the maternal-fetal interface on trophoblasts and uterine immune cells [17]. It is likely that differential uterine immune responses occur due to the diversity of pathogens that ensues activation of any one of the TLRs, ultimately leading to deleterious inflammation and preterm birth. 

This paper aims to summarize specific viral and bacterial pathogens that may program preterm birth outcomes. Furthermore, we expound upon possible routes of transmission and areas of replication that different foreign organisms may home to. Finally, after a brief discussion of the necessary steps toward an inflammatory environment that programs term labor, we elaborate on pathogenic triggers of this process that may activate TLRs to induce preterm birth.

## 2. Keys to Parturition

In order to delineate the aberrant induction of inflammation by infectious agents, the role of inflammation in healthy pregnancy outcomes must be understood. The act of birth is characterized by the onset of uterine contractions that lead to expulsion of the fetus from the uterine cavity. The act of giving birth is the ultimate step in a proinflammatory signaling cascade that is orchestrated by an intrauterine milieu coupled to hormonal cues.

Temporal increase in inflammatory signals initiates labor. Inflammatory cytokines and chemokines such as TNF-*α*, IL-1*β* and IL-8 increase in the placental microenvironment, including amniotic fluid and fetal membranes. This induces signals for innate immune cells to become activated [18, 19]. Upon initiation of a proinflammatory cascade including NF-*κ*B activation, uterine immune cells produce inflammatory chemokines and cytokines. Increased uterine activation of transcription by NF-*κ*B leads directly to high levels of COX-2, PGE_2_, the gap junction protein connexin 43, and upregulation of the oxytocin receptor [20]*.* The increase of the aforementioned proteins and receptors leads to active uterine stretching and thus to the induction of birth.

The pregnant uterus is replete with specialized immune cells primed to play roles in implantation, placentation, and parturition. The major cell types studied thus far are uterine NK cells (uNK), dendritic cells, T regulatory cells (Treg), and macrophages. uNK cells are the most thoroughly studied uterine immune cells due to lack of their natural cytotoxicity, an alternative receptor repertoire to peripheral blood counterparts, and the ability to produce vascular growth factors, cytokines, and chemokines during early stages of pregnancy [21, 22]. In humans, uNK cells are reduced after the second trimester. This trend is also followed in mice. However, uNK cells can be amplified in mice in response to inflammatory triggers at later stages of pregnancy. It is thus tempting to speculate that such a phenomenon may also occur in humans. Research has expanded to investigate the ability of uNK cells to create a balanced milieu at the maternal-fetal interface due to their ability to produce IL-10, IP10, and IL-8 during cross-talk with trophoblasts and dendritic cells [23–35]. uNK cells are found interwoven throughout decidual areas where spiral arteries are bountiful. This positioning is demonstrative of their role in growth and decidualization of the placental organ [22]. uNK cells peak in humans in first trimester and their numbers start to decrease at the end of second trimester. Mouse models deficient in uNK cells show that proper birth takes place in the absence of these cells; however, the placentation that occurs is shallow and less developed than models where uNK cells are available [26]. While uNK cells are not thought to play a direct role in term birth, several murine studies have shown that these cells can become cytotoxic in nature in the presence of certain pathogens and ultimately lead to preterm birth through production of TNF-*α* [13, 14]. Thus, it stands to reason that the disappearance of uNK cells in mid-third trimester is a fail-safe mechanism for the course of term birth that allows the uterus to minimize unscheduled inflammatory events.

While uNK cells do not play a documented role in term birth, macrophages may hold the key to conduction of proper parturition. Uterine macrophages are found in proportions upwards of 20% of the total uterine lymphocyte population. Over the course of pregnancy, uterine macrophages are present as innate immune sentinels due to their expression of TLRs. However, macrophages are immunomodulatory over the course of gestation as they simultaneously produce TNF-*α*, IL-10, and TGF-*β* throughout placentation [27]. Along similar lines, matrix metalloproteinases (MMPs) are secreted in early and late gestation for tissue degradation which in turn induces MIP-1*α* and MCP-1 to signal the further invasion of macrophages for phagocytosis [28–30]. Reports show that late stage stimulation of uterine macrophages induces high levels of IL-8 production, a neutrophil and macrophage chemoattractant. In line with these findings, the act of parturition itself is associated with high levels of macrophage and neutrophil infiltration as these two cell types can quickly engulf, remove, and remodel tissue [31].

The function and phenotype of dendritic cells in the uterus is beginning to be expounded upon. Recent studies have highlighted their role in crosstalk with uNK cells and trophoblasts in order to orchestrate production of cytokines such as TNF-*α*, IL-12, and IL-10 [32]. While the role of DCs in parturition remains unknown, it is fair to assume that these cells add to the balanced cytokine and signaling milieu required throughout gestation. Furthermore, recent characterization in mice of a uterine-specific subset of DCs that is skewed toward production of IL-15 and CCL6 adds to evidence that their actions are dichotomous [33]. Thus, while these cells contribute to an immunosuppressive atmosphere over the course of gestation, they possess the capacity to contribute to proinflammatory responses upon pathogenic activation.

While uNK cells, macrophages, and dendritic cells aid to orchestrate the balance between pro- and anti-inflammatory milieu over the course of gestation, T regulatory cells Tregs in the uterus are thought to be mainly immunosuppressive. Studies demonstrate the importance of these cells as adoptive transfer of Tregs into abortion prone mice leads to pregnancy rescue [34]. T-cell-deficient Rag1^−/−^ mice given the TLR4 agonist LPS deliver preterm. Infusion of Tregs into these mice rescues pregnancy to term, indicating an advantageous role for these cells in TLR-pathogen mediated response to adverse pregnancy outcomes [35]. The function and phenotype of T regs found in the uterus remains unknown, but these cells are presenting as a possible therapeutic cell to aid in rescue of pregnancy loss. 

While the aforementioned cellular activities are highly orchestrated throughout pregnancy and particularly during the onset of labor, these actions are correlated to specific hormonal cues that allow for these changes. For the majority of pregnancies one of the main mechanisms that allows for uterine quiescence and lack of uterine muscle movement is the increased presence of the hormone progesterone [36, 37]. Female sex hormones have been shown to decrease the T-cell stimulatory capacity of DCs during pregnancy [38]. In rodents, direct treatment of DCs with progesterone has been shown to increase the inhibitory capacity of DCs and these changes are associated with progesterone receptor (PR) regulation. Due to the fact that human and murine uNK cells do not possess PRs, the capacity of DCs to produce IL-15 for uNK development may work through indirect mediation of DCs by progesterone [39, 40]. Importantly, macrophages possess PR and are directly regulated by progesterone as their migration and nitric oxide production is severely impaired when progesterone levels are high. In support of these findings, PR^*‒*/*‒*^ mice show high levels of macrophage and neutrophil infiltration in the pregnant uterus [3]. Taken together, the functional withdrawal of progesterone is critical to the initiation of parturition as its decreased action allows for control of immune cells needed to initiate inflammatory cascades to be relinquished.

## 3. Preterm Birth: Activated Uterine Immunity

How does the presence of viral or bacterial organisms lead to preterm birth? It is rarely the foreign organism itself that causes preterm birth. Rather, it is the immune response of the host evoked by the pathogen that leads to aberrant pregnancy outcomes. With the knowledge of parturition as a process mediated by inflammation, we can begin to understand that pathogenic insults can trigger unscheduled uterine immunity. Evidence demonstrates that the activated TLR pathways and the route of pathogenic entry may determine the immunological cascade of aberrant cellular and cytokine activity that lead to preterm birth ([11–15], our unpublished data). The majority of these pathways commonly lead to increased NF-*κ*B activity that allows for production of inflammatory cytokines and mediation of progesterone withdrawal through modulation of the participation of different PRs [20]. 

Briefly, the immune system at the maternal-fetal interface thus far has been discussed due to its virtue as specialized toward growth and decidualization of the placental organ and delicately regulated by sex hormones. However, new observations have deciphered that innate immune defenses, characteristic of innate immunity on peripheral blood cells, are present at the placental level as well. The TLRs are expressed at the placental level on trophoblasts, uNK cells, DCs, and macrophages [17, 21]. Activation of any of the TLRs leads to a downstream cascade of proinflammatory cytokine production, and most notably, to activation of the NF-*κ*B transcription factor. It stands to reason that TLR activation at the maternal-fetal interface may tip the tightly regulated cytokine and hormonal anti-inflammatory milieu necessary for successful term pregnancy. 

**Figure 1:**
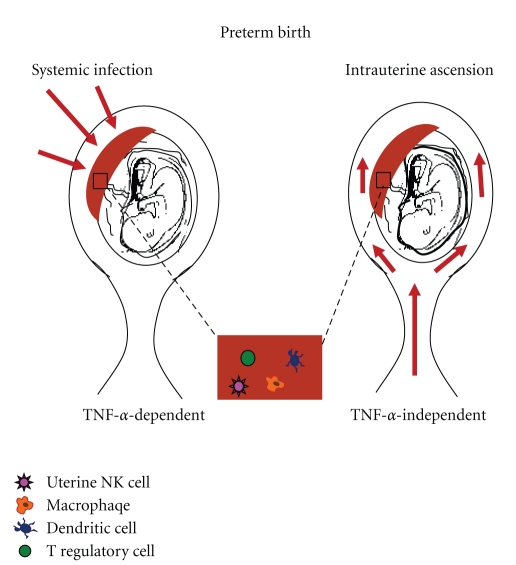
*Roads to Preterm*. Birth pathogens may enter the placental blood supply via systemic circulation due to previous or current onset of infection. Murine models where intraperitoneal injections are employed are utilized to mimic maternal systemic infection in order to study the ability of a pathogen to activate decidual immunity. Data presented in this paper demonstrate that systemic infections correlate to TNF-*α* -dependent immune responses that ultimately induce preterm birth. In contrast, intrauterine ascending infections occur when a pathogen ascends the uterine cavity via the vaginal tract. Surgical procedures in mice, rats, and rabbits have mimicked uterine cavity infections through intrauterine infusion of pathogens directly into the amniotic sac or between two placental units. Importantly, data summarized here demonstrate that pathogens introduced through intrauterine ascension do not tend to activate a TNF-*α* -driven axis to induce preterm birth.

Two forms of pathogenic entry are studied in rodent models due to their correlations with human infection. Intrauterine ascension through the vaginal tract and systemic infiltration are mimicked by intrauterine infusion and intraperitoneal injections, respectively (Figure 1). Bacteria show stronger correlations throughout the literature with increased incidence of preterm birth than viruses. This may be due to the differential sites of infection that bacteria versus viruses target. Generally, bacteria are found in mucosal membranes that surround the amniotic sac or those lining the intrauterine canal [8]. On the other hand, reports demonstrate that viruses, in need of host cell machinery for replication, tend to infect trophoblast cells of the placenta as these cells possess specific receptors needed for viral particle entry [41–43]. Importantly, bacteria are easily cultured from amniotic fluid or from swab samples taken from the uterine tracts of pregnant women [7, 44]. Due to the nature of viruses, these pathogens are more difficult to pinpoint, though some studies have been able to isolate HIV and adenovirus from amniotic fluid samples [8, 45].

## 4. Bacterial Entry

Bacterial infections manifest in the pregnant uterus generally between maternal and fetal membranes or directly within amnion or chorion specific fetal membranes. Infection of the amniotic cavity is denoted amnionitis, direct infection of fetal membranes is denoted chorioamnionitis; whereas funisitis is infection of the umbilical cord. Interestingly, several reports note that bacterial agents are rarely found at the placental level, in contrast to viral pathogens [7, 8, 45]. Due to the fact that bacteria can subsist extracellularly within membranes, it is reasonable to postulate that intrauterine bacteria or fetal DNA due to necrosis of fetal cells are visualized by TLRs that are extracellular such as TLR2, TLR4, TLR5, and TLR6 [46, 47].

Effective methods of probing for the presence of bacterial agents are immunostaining of membranes of the chorion or amnion from term versus preterm deliveries. Membranes from preterm delivered placentas show higher levels of chemotactic proteins MCP-1 and CXCL6 (Granulocyte chemotactic protein-2), inflammatory cytokines IL-1*β*, TNF-*α*, and COX-2 by immunohistochemical staining [48–50]. Furthermore, the presence of bacterial agents can be probed for by testing the amniotic fluid for microbial invasion of the amniotic cavity (MIAC). Women undergoing preterm labor with premature rupture of membranes (PROM) show 34% positive MIAC pathology. Interestingly, at the time that these women deliver 75% show positive MIAC pathology. It has been suggested that the inflammatory atmosphere of preterm labor allows for increased bacterial proliferation [44, 51].

## 5. Viral Entry

Though data is relatively scarce, evidence suggests that viral entry into trophoblast cells induces trophoblast apoptosis and the resultant inflammatory events can lead to preterm birth [52]. The general route of entry in placental viral infections is cell-specific as different viruses enter trophoblasts that express their viral receptor [42, 52, 53]. Once viral uncoating or shedding occurs within a cell, intracellular TLRs are available as sentinels within endosomal pockets where viruses may replicate. Intracellular TLRs, TLR3, TLR7/8, and TLR9 are specific for viral genomic motifs and, not surprisingly, are associated with several viruses that prove correlation with adverse pregnancy outcomes.

## 6. TLRs: Sentinels of Uterine Immunity

Here, we summarize known pathogens, viral or bacterial, associated with induction of preterm birth. We highlight immunological pathways that demonstrate specific PAMPs that activate TLRs to induce preterm birth. Work in mouse models illustrates differential TLR-mediated cascades of inflammatory-based uterine immunity that can lead to preterm birth. Importantly, clinical correlates where amniotic fluid is tested or maternal/fetal membranes are probed begin to support these findings.

### 6.1. TLR2

TLR2 is one of the more promiscuous TLRs in that upon activation it can concomitantly signal with TLR1 and TLR6. In line with these findings, TLR2 is not only specific for gram positive bacteria due to its specificity for peptidoglycan, but also signals upon recognition of meningococcal porins, fungal, parasitic, and viral pathogen-associated molecular patterns [12]. Studies show that women who deliver preterm with the condition of chorioamnionitis show significant upregulation of the TLR2 receptor as compared to controls, implicating a role for the receptor in preterm birth and infection [54]. Murine studies demonstrate preterm birth occurs when TLR2 is activated either through systemic or intrauterine delivery ([11], our unpublished data). Systemic activation induces activation of the NF-*κ*B transcription factor, and first trimester trophoblasts produce significant amounts of IL-8 and IL-6 ultimately undergoing apoptosis [12]. In contrast, intrauterine delivery of TLR2 agonists did not induce increased TNF-*α* production or NF-*κ*B activation [11] (Table 1).

TLR2-specific human pathogens that correlate with increased incidence of preterm birth are *ureaplasma ureaticulum*, group B *streptococcus*, and cytomegalovirus (CMV) [55, 56]. *Ureaplasma ureaticulum* is one of three bacteria that are grouped together and termed bacterial vaginosis. While common genital infections such as *Chlamydia trachomatis* and *neisseria gonorrhoea* are rarely associated with aberrant pregnancy outcomes, high loads of bacterial vaginosis in pregnant women prove nine times more likely to lead to prematurity [47]. Thus, the activity of TLR2 in the presence of *ureaplasma ureaticulum* is an important correlation in the literature to follow at the clinical level. CMV shows the greatest correlation of human infection and adverse pregnancy outcomes as this herpes virus is present in 80% of the general population, but becomes active under conditions of immune suppression. Data demonstrate that human CMV may infect syncitiotrophobalsts or cytotrophoblasts and induce proinflammatory cascades such as IL-8 production [57, 58].

### 6.2. TLR3

While TLR2 generally “sees” pathogens that are extracellular, TLR3 possesses specificity for double stranded RNA viral motifs and is found in endosomal pockets. Recently, it was shown that TLR3 can recognize products of cellular necrosis, a fact of importance for a system such as pregnancy where cellular turnover is abundant [59]. Evidence suggests that TLR3 activation in trophoblasts may be a key mechanism to preterm birth outcome induced by infectious agents [60]. 

Trophoblasts express the coxsackievirus and adenovirus receptor (CAR). Upon adenoviral infection, trophoblasts underwent apoptosis and this led to recruitment of a decidual immune response [42]. Studies in human and mouse trophoblast cells show that cells were treated with synthetic TLR3 ligand poly(I:C), and the NF-*κ*B pathway was activated leading to downstream cascades of inflammatory signals. Studies have utilized poly(I:C) as well to assess the role of preterm birth through TLR3 activation in mouse models. Where poly(I:C) was injected intraperitoneally to mimic a maternal systemic infection, mice gave preterm birth through an NK-*κ*B-dependent mechanism and demonstrated marked serum and placental production of IL-6, IL-12, and MCP-1 [11, 60]. Our unpublished results demonstrate that preterm birth in WT mice is induced by a TNF-*α*-mediated axis. In contrast, in a model of intrauterine infusion, INF-*β*  and CCL5 were the major contributors to preterm birth outcomes in response to poly(I:c) and TNF-*α* production was not significant [11]. Taken together, these studies lend evidence to the hypothesis that route of entry of a virus may play a role in the specificity of the immune response evoked (Table 1).

### 6.3. TLR4

It is well established that TLR4 recognizes lipopolysacharride motifs found on the majority of gram negative bacteria. Two of the three bacterial strains associated with preterm birth outcomes, *mycoplasma hominis* and *trachtomonas vaginalis,* are gram negative and thus have the capacity to signal via TLR4 [46]. Activation of TLR4 at the placental level has been studied in order to assess the possible immune activation that ensues when TLR4 specific bacterial loads overwhelm the uterine cavity and trigger preterm birth [61]. Our lab has demonstrated that LPS and *E. coli *cause increased uNK cell cytotoxicity and TNF-*α* production, ultimately leading to preterm birth. Neutralization of TNF-*α*  or abolition of uNK cells rescued pregnancy to term. In contrast, our unpublished model of *E. coli* intrauterine infusion demonstrates that preterm birth does occur, but not in a TNF-*α*-dependent manner ([13], our unpublished data). Similar unpublished data from our lab agree with this data as the NF-*κ*B  pathway was not activated in a model of preterm birth mediated by LPS intrauterine infusion (Table 1). Again, these results present strong evidence for the hypothesis that alternative routes of pathogenic entry, for both viral and bacterial pathogens, can cause markedly different immune responses, both ultimately leading to preterm birth.

### 6.4. TLR7/8

TLR7/8 are specific for single stranded RNA motifs, thus retroviruses are strong candidates to activate these receptors. Though HIV is not yet correlated to a specific TLR for activation, evidence suggests that TLR7 activation mimics HIV related pathologies [62]. Importantly, strong associations exist between HIV seroprevalance and incidence of preterm birth. A cohort study that followed 600 women, approximately half seropositive for HIV and half seronegative, showed that HIV^+^ women were significantly more likely to give preterm birth. Furthermore, the seropositive women who gave preterm birth showed significantly diminished levels of CD4^+^ T cells [63]. Taken together, these data demonstrate that either HIV or the immune-compromised state of mothers may lead to increased risk of preterm birth. 

Studies in nonpregnant patients aim to activate TLRs in order to discourage HIV activity due to the proinflammatory activity of antiretroviral therapy [64]. Taken together, the immunosuppressive state of pregnancy needs to be thoroughly considered when therapy is employed in pregnant women, but lessons may be learned from how the virus travels and replicates during this state.

### 6.5. TLR9

TLR9 recognizes unmethylated CpG motifs. Though reports identify this intracellular receptor as specific for double stranded DNA viruses or fetal DNA, these types of CpG motifs constitute over 80% of bacterial genomes as well. Studies that activate TLR9 with CpG in pregnant mice thus far have demonstrated that a lack of IL-10 causes high susceptibility to CpG-mediated pathogenic mimics and inflammatory responses composed of severe placental macrophage and neutrophil influx coupled to TNF-*α* production and preterm birth [15] (Table 1).

The family of herpes viruses is associated with TLR9 activation and reports demonstrate that active viral shedding during pregnancy is associated with preterm birth outcomes. Importantly, since herpes viruses are latent or chronic, if the virus is inactive during the term of pregnancy there are no significant findings that viral carriers have adverse pregnancy outcomes [65]. While cytomegalovirus belongs to the herpes virus family, it has been associated with activation of TLR2 in humans, while murine CMV is specific for TLR9. Mouse models demonstrate that CpG motifs specific for TLR9 activation do lead to adverse pregnancy outcomes in IL-10^*‒*/*‒*^ mice [15]. In wild type mice, the adverse effects were on nonlive born pups that experienced cranial-facial and distal limb malformation [66]. Interestingly, infants born to CMV-infected mothers have shown similar malformations [67].

## 7. Conclusion

Taken together, the aforementioned studies and resultant data demonstrate that pathogenic route of entry as well as the specific TLR activated may be two important determinants of the immune responses that induce preterm birth. Murine studies have begun to delve into the role of NF-*κ*B and its direct role in TNF-*α* production. Importantly, an amassment of current literature seems to suggest that systemic inflammatory responses may induce TNF-*α* through the NF-*κ*B pathway to activate events leading to preterm birth. In contrast, in the intrauterine setting the mode of action is most likely TNF-*α*-independent. Therefore, more research needs to occur to better understand the underlying inflammatory or hormonal imbalances that are induced by intrauterine infection that lead to preterm birth outcomes. 

In regards to activation of specific TLRs, systemic injection of different PAMPs demonstrates that alternative immune cell subsets respond based on the pathogen at play. Bacterial pathogens signaling through TLR2 or TLR4 show strong staining within human chorion and amnion membranes. Murine studies emphasize the role of uNK cell amplification and TNF-*α* production in response to TLR4 activation. TLR3 activation data from our lab demonstrates, in WT mice, that uNK cells amplify and produce TNF-*α* in response to poly(I:C), a viral mimic. Importantly, other reports show that systemic injection of poly(I:C) induces trophoblast apoptosis after a vigorous cytokine storm lending to a preterm birth outcome. Taken together, these two reports are not disparate. Finally, in the context of TLR9 activation it has been shown that macrophages and neutrophils infiltrate the placental zone and lead to preterm birth or intrauterine fetal death outcomes. 

As a whole, the reports discussed in this paper lend strong evidence to the postulate that the pathway to induction of preterm birth is dependent on the TLR activated—a product of the type of pathogenic infection. Secondly, the route of entry, intrauterine ascension versus systemic infection, plays a role in determining the cytokine profile activated and the ensuing mechanism of fetal rejection. From a clinical standpoint, it may be paramount to account for not only the type of infectious agent at play within the maternal-fetal system, but also the route through which the pregnancy was compromised.

## Figures and Tables

**Table 1:** Routes of pathogenic entry coupled to specific TLR activations lead to preterm birth via NF-*κ*B-TNF-*α* -dependent and independent pathways.

TLR	(PAMP)	Route of Entry	NF-*κ*B	TNF-*α*	Pregnancy Outcome
TLR2	peptidogylcan	IU	−	−	Preterm Birth^11^

TLR3	poly(I:C)	IU	−	−	Preterm Birth^11, up^
TLR3	poly(I:C)	Systemic	+	+	Preterm Birth^60^

TLR4	LPS/*E. coli *	IU	N/A	−	Preterm Birth^up^
TLR4	LPS/*E. coli *	Systemic	N/A	++	Preterm Birth^13^

TLR9	CpG	IU	N/A	−	Preterm Birth/IUFD^up^
TLR9	CpG	Systemic	N/A	++	Preterm Birth/IUFD^15^

– ^up^ is our unpublished data
